# Calmodulin antagonists induce cell cycle arrest and apoptosis in vitro and inhibit tumor growth in vivo in human multiple myeloma

**DOI:** 10.1186/1471-2407-14-882

**Published:** 2014-11-26

**Authors:** Shigeyuki Yokokura, Saki Yurimoto, Akihito Matsuoka, Osamu Imataki, Hiroaki Dobashi, Shuji Bandoh, Takuya Matsunaga

**Affiliations:** Department of Internal Medicine, Division of Hematology, Rheumatology and Respiratory Medicine, Faculty of Medicine, Kagawa University, 1750-1 Ikenobe, Miki-cho, Kita-gun, Kagawa, 761-0793 Japan

**Keywords:** Calmodulin, Multiple myeloma, Cell cycle, Apoptosis

## Abstract

**Background:**

Human multiple myeloma (MM) is an incurable hematological malignancy for which novel therapeutic agents are needed. Calmodulin (CaM) antagonists have been reported to induce apoptosis and inhibit tumor cell invasion and metastasis in various tumor models. However, the antitumor effects of CaM antagonists on MM are poorly understood. In this study, we investigated the antitumor effects of naphthalenesulfonamide derivative selective CaM antagonists W-7 and W-13 on MM cell lines both in vitro and in vivo.

**Methods:**

The proliferative ability was analyzed by the WST-8 assay. Cell cycle was evaluated by flow cytometry after staining of cells with PI. Apoptosis was quantified by flow cytometry after double-staining of cells by Annexin-V/PI. Molecular changes of cell cycle and apoptosis were determined by Western blot. Intracellular calcium levels and mitochondrial membrane potentials were determined using Fluo-4/AM dye and JC-10 dye, respectively. Moreover, we examined the in vivo anti-MM effects of CaM antagonists using a murine xenograft model of the human MM cell line.

**Results:**

Treatment with W-7 and W-13 resulted in the dose-dependent inhibition of cell proliferation in various MM cell lines. W-7 and W-13 induced G1 phase cell cycle arrest by downregulating cyclins and upregulating p21^cip1^. In addition, W-7 and W-13 induced apoptosis via caspase activation; this occurred partly through the elevation of intracellular calcium levels and mitochondrial membrane potential depolarization and through inhibition of the STAT3 phosphorylation and subsequent downregulation of Mcl-1 protein. In tumor xenograft mouse models, tumor growth rates in CaM antagonist-treated groups were significantly reduced compared with those in the vehicle-treated groups.

**Conclusions:**

Our results demonstrate that CaM antagonists induce cell cycle arrest, induce apoptosis via caspase activation, and inhibit tumor growth in a murine MM model and raise the possibility that inhibition of CaM might be a useful therapeutic strategy for the treatment of MM.

## Background

Multiple myeloma (MM) is a hematological malignancy characterized by the excess accumulation of plasma cells in the bone marrow and the production of monoclonal immunoglobulins or paraproteins [1]. Despite conventional therapies including alkylating agents, anthracyclines, and corticosteroids [2, 3] as well as intensive therapies, including autologous hematopoietic stem cell transplantation [4] and the novel agents bortezomib, thalidomide, and lenalidomide [5–7], the incurable nature of MM continues to stimulate the investigation of novel drugs.

Calmodulin (CaM), an ubiquitous intracellular calcium-sensing protein, mediates the effects of changes in the cytoplasmic Ca^2+^ level and is involved in the regulation of many biological processes. In particular, CaM has been shown to play important roles in cell cycle progression and apoptosis regulation. In cell cycle progression, the concentration of CaM progressively increases, reaches high levels at the G1/S transition, and remains high during the ensuing progression of the cell cycle. In apoptosis regulation, CaM regulates apoptotic processes both positively and negatively mediating elevated intracellular Ca^2+^, which can have both growth promoting and cell death-inducing consequences [8]. CaM has also been reported to be highly expressed in mRNA level in MM cells compared with normal plasma cells in the identical twins study [9], and the mRNA expression level of CaM has shown to be higher in plasma cells in the patients of monoclonal gammopathy of undetermined significance compared with normal plasma cells [10]. There is evidence that specific antagonists of CaM inhibit the growth of a variety of tumor cells, such as lung cancer cells, breast cancer cells, and cholangiocarcinoma cells [11–13]. CaM antagonists also reduce cell invasion in human melanoma cell lines [14] and Lewis lung carcinoma-induced lung metastasis [15]. However, neither the in vitro nor in vivo antitumor effects of CaM antagonists on MM are well understood.

In this study, we investigated the effects of the naphthalenesulfonamide derivatives W-7 and W-13, selective and cell-permeable CaM antagonists, on proliferation, cell cycle progression, and apoptosis in human MM cell lines. Furthermore, we demonstrated that CaM antagonists inhibited human MM tumor growth in xenografted mouse models. These studies suggest that inhibition of CaM might be a potential therapeutic strategy for MM treatment.

## Methods

### Antibodies and reagents

Rabbit anti-cyclin D1 polyclonal antibody, rabbit anti-cyclin D2 (D52F9) monoclonal antibody, mouse anti-cyclin E1 (HE12) monoclonal antibody, rabbit anti-cyclin-dependent kinase (CDK) 2 (78B2) monoclonal antibody, mouse anti-CDK4 (DCS156) monoclonal antibody, mouse anti-CDK6 (DCS83) monoclonal antibody, mouse anti-retinoblastoma protein (Rb) (4H1) monoclonal antibody, rabbit anti-phospho-Rb (Ser795) polyclonal antibody, rabbit anti-p21^cip1^ (12D1) monoclonal antibody, rabbit anti-p27^kip1^ polyclonal antibody, rabbit anti-caspase-9 polyclonal antibody, rabbit anti-cleaved caspase-9 (Asp330) polyclonal antibody, rabbit anti-caspase-8 (D35G2) monoclonal antibody, rabbit anti-cleaved caspase-8 (Asp391) monoclonal antibody, rabbit anti-caspase-3 polyclonal antibody, rabbit anti-cleaved caspase-3 (Asp175) monoclonal antibody, rabbit anti-caspase-7 polyclonal antibody, rabbit anti-cleaved caspase-7 (Asp198) polyclonal antibody, rabbit anti-PARP polyclonal antibody, rabbit anti-STAT3 (79D7) monoclonal antibody, rabbit anti-phospho-STAT3 (Tyr705) polyclonal antibody, and rabbit anti-Mcl-1 polyclonal antibody were obtained from Cell Signaling Technology (Beverly, MA) and mouse anti-GAPDH (0411) monoclonal antibody, rabbit anti-ERK1/2 (H-72) polyclonal antibody, and goat anti-phospho-ERK1/2 (Thr 202/Tyr204) polyclonal antibody were obtained from Santa Cruz Biotechnology (Santa Cruz, CA). Rabbit anti-calmodulin (Ab-79/81) polyclonal antibody was purchased from Assay Biotechnology Company Inc. (Sunnyvale, CA) W-5, W-7, and W-13 were purchased from Tokyo Kasei Industry (Tokyo, Japan).

### Cells and cell culture

Human MM cell lines RPMI 8226, U266, MM1.S, and MM1.R were purchased from the American Type Culture Collection (ATCC, Manassas, VA), and KMS-5, KMS-12-BM, and NCI-H929 lines were kindly provided by Dr. Kensuke Matsumoto (Institute of Internal Medicine, Faculty of Medicine, Kagawa University, Japan). All cell lines were recharacterized by short tandem repeat profiling to confirm no cross-contamination. All cell lines except KMS-12-BM and NCI-H929 were maintained in RPMI 1640 medium (Life Technologies, Carlsbad, CA) with 10% fetal bovine serum (FBS; Life Technologies), 100 U/mL of penicillin (Wako, Osaka, Japan), and 100 μg/mL of streptomycin (Sigma-Aldrich Corporation, St. Louis, MO) and were cultured at 37°C. For the KMS-12-BM and NCI-H929 lines, 0.05 mM of 2-mercaptoethanol was added to the culture medium described above.

### Cell proliferation assay

MM cells were seeded in a 96-well plate at a density of 20,000 cells/well in 100 μL of culture medium; the cells were maintained at 37°C for 24 h in the presence of various concentrations (0–80 μM) of W-5, W-7, or W-13. This culture step was followed by 3 h incubation with 10 μL of WST-8 labeling reagent (Cell Counting Kit-8; Dojindo, Kumamoto, Japan), after which the absorbance at 450 nm was read on a microplate reader.

### Cell cycle analysis

MM cells (1 × 10^6^/well) were cultured in the presence or absence of CaM antagonists for 24 h. The cells were washed, fixed in ethanol for 2 h, and stained with propidium iodide using a Cell Cycle Phase Determination Kit (Cayman Chemical, Ann Arbor, MI) according to the manufacturer’s protocol. The samples were analyzed on a Cytomics FC 500 flow cytometer (Beckman Coulter) with a 488-nm excitation laser. Live cells were gated according to the forward and side scatter profiles. The percentage of cells in each phase of the cell cycle was calculated using MultiCycle AV software (Phoenix Flow Systems, San Diego, CA).

### Apoptosis assay

MM cells (1 × 10^6^/well) were treated with or without CaM antagonists for 24 h. The cells were then incubated with FITC-annexin V and propidium iodide (Alexa Fluor 488 Annexin V/Dead Cell Apoptosis kit; Life Technologies, Eugene, OR) according to the manufacturer’s protocol. Apoptosis was subsequently assessed by flow cytometry. Using flow cytometric analysis plots of cells with annexin-V on the x-axis and propidium iodide on the y-axis, the percentages of the cell population were determined for each of the following quadrants: lower left, normal cells; lower right, early apoptotic cells; upper right, late apoptotic and necrotic cells.

### Western blot analysis

For each condition, 5 × 10^6^ cells were cultured with or without CaM antagonists for 24 h. The cells were lysed in RIPA buffer (Thermo Scientific, Rockford, IL) in the presence of protease and phosphatase inhibitor cocktail (Thermo Scientific). The proteins were separated on an acrylamide gel and transferred onto a polyvinylidene difluoride membrane (Bio-Rad, Hercules, CA). The membranes were then blocked for 1 h in PBS containing 5% non-fat dried milk and 0.05% Tween-20, followed by an incubation of several hours with primary antibodies. The membranes were washed in PBS–Tween-20 buffer and incubated with the appropriate HRP-conjugated secondary antibody. The membranes were visualized by chemiluminescence using enhanced chemiluminescence reagents (GE Healthcare, Little Chalfont, Buckinghamshire, UK). GAPDH was detected as a protein loading control.

### Measurement of intracellular Ca^2+^ levels

Washed MM cell lines were loaded with 4 μM of fluorochrome fluo-4-acetoxymethyl ester (Fluo-4/AM; Dojindo, Kumamoto, Japan) in PBS for 1 h at 37°C in the dark. After washing, the cells were resuspended at a concentration of 1 × 10^6^ cells/mL. The external Ca^2+^ level was adjusted to 1 mM, and the dyed cells were incubated with 60 μM of CaM antagonists at 37°C for 15 min in the dark and analyzed by flow cytometry.

### Detection of mitochondrial membrane potential depolarization

A total of 1 × 10^6^ cells were loaded with 10 μg/ml of JC-10 (Cell Meter™ JC-10 Mitochondrial Membrane Potential Assay Kit; ABD Bioquest Inc., Sunnyvale, CA) at 37°C for 30 min. Next, the cells were treated with 60 μM of CaM antagonists at 37°C for 1 h and the visualized using a fluorescence microscope (Olympus BX-51/DP-72; Olympus, Tokyo, Japan) fitted with a WIB filter (excitation, 460–490 nm; dichroic mirror, 505 nm; emission barrier filter, 510 nm).

### In vivo treatment with CaM antagonists on the RPMI 8226 mouse model

Six-week-old female BALB/c *nu* mice were purchased from Charles River Japan (Atsugi, Japan). The animals were housed under specific pathogen-free conditions and had free access to food and tap water. All procedures involving these mice were approved by the local animal ethics committee at Kagawa University. The mice were inoculated subcutaneously in the flank with 1 × 10^7^ RPMI 8226 cells. Seven days after injection, the mice were randomly divided into two comparison groups with 10 mice each to ensure proper controls for both agents. Because W-7 forms insoluble deposits in PBS, it was dissolved in water. The comparison groups were the vehicle (H_2_O, n = 5) vs. W-7 (dissolved in H_2_O, n = 5) group and the vehicle (PBS, n = 5) vs. W-13 (dissolved in PBS, n = 5) group. The mice were injected intraperitoneally with H_2_O, W-7 (3 mg/kg), PBS, or W-13 (3 mg/kg) on 5 consecutive days per week. The tumor sizes were measured twice weekly in two dimensions using calipers, and the tumor volume was calculated using the formula V = 0.5 (a × b^2^), where a is the long diameter of the tumor and b is the short diameter of the tumor. The animals were sacrificed when the tumor diameters reached 2 cm or became ulcerated. After treatment completion, the xenografts or selected organs (heart, lung, kidney, liver, and pancreas) were excised, fixed in formalin, embedded in paraffin, and cut into 5.0 μm sections. Adjacent sections were stained with hematoxylin and eosin (H&E) or subjected to a terminal deoxyribonucleotide transferase–mediated nick-end labeling (TUNEL) assay (ApopTag In Situ Apoptosis Detection Kit; Intergen, Purchase, NY). The apoptotic index was calculated as the number of TUNEL-positive cells divided by the total number of cells in 10 randomly selected high-power fields.

### Statistical analysis

All values were expressed as means ± standard deviations. The statistical differences between groups were determined using paired Student’s *t* tests. A *P* value of <0.01 was considered significant.

## Results

### Calmodulin inhibitors inhibits MM cell proliferation in vitro

**Figure 1:**
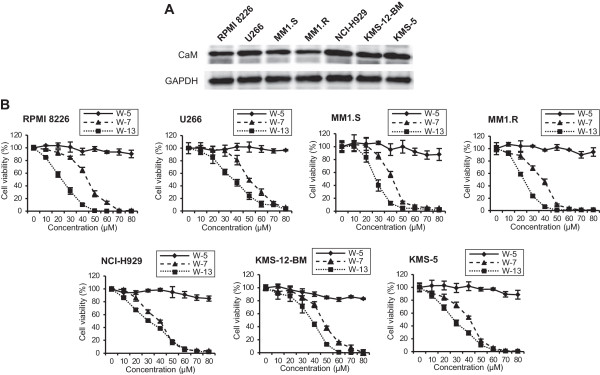
**Effects of CaM antagonists on human multiple myeloma cell proliferation. (A)** The basal protein expression levels of CaM in the multiple myeloma cell lines RPMI 8226, U266, MM1.S, MM1.R, NCI-H929, KMS-12-BM, and KMS-5 were determined by western blot analysis. **(B)** Cells were treated with 0–80 μM W-5, W-7, and W-13 for 24 h, after which cell proliferation was assayed according to the WST-8 method.

**Figure 2:**
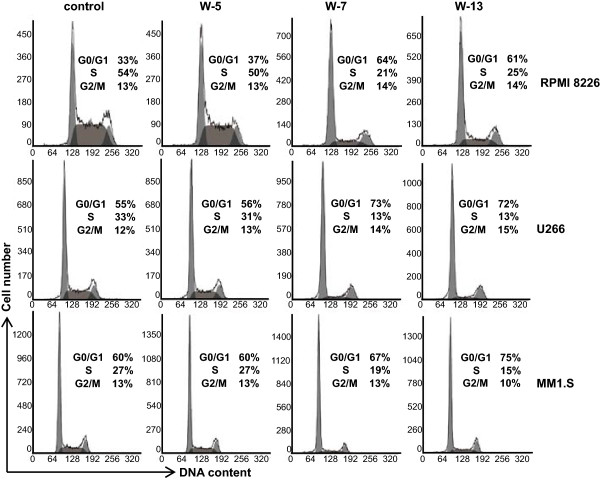
**Effects of CaM antagonists on the cell cycle in human multiple myeloma cells.** Cells were incubated with CaM antagonists (40 μM). After 24 h, the cells were stained with propidium iodide and analyzed by flow cytometry. Live cells were gated according to their forward scatter/side scatter parameters.

**Figure 3:**
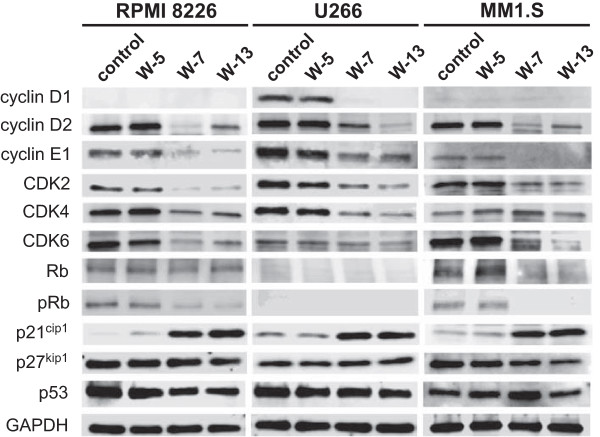
**Effects of CaM antagonists on the expression of various cell cycle regulatory proteins.** Cells were treated with CaM antagonists (40 μM) for 24 h and then lysed. The lysates were analyzed by western blotting with the indicated antibodies.

**Figure 4:**
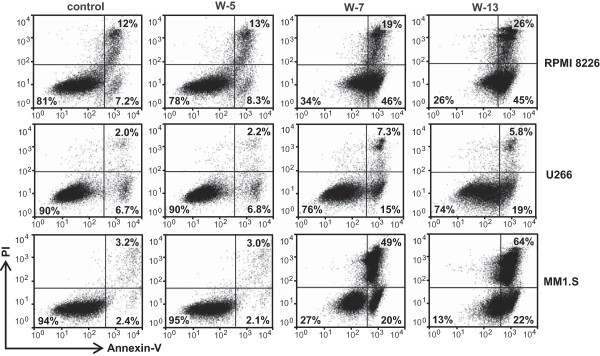
**Effects of CaM antagonists on apoptosis in multiple myeloma cells.** Cells were treated with CaM antagonists (60 μM) for 24 h. The cells were subsequently harvested and incubated with FITC-labeled annexin V and propidium iodide and analyzed by flow cytometry. In the representative flow plots, the lower left quadrant contains normal cells; the lower right quadrant contains early apoptotic cells; and the upper right quadrant contains late apoptotic and necrotic cells.

**Figure 5:**
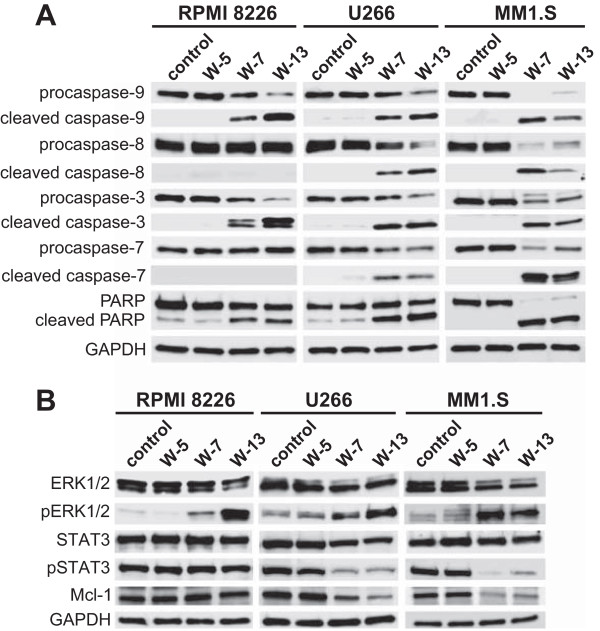
**Effects of CaM antagonists on apoptosis-related proteins and intracellular signaling proteins.** Cells were treated with CaM antagonists (60 μM) for 24 h and then lysed. The lysates were analyzed by western blotting using the indicated antibodies against proteins related to caspase-dependent apoptosis **(A)** and intracellular signaling **(B)**.

**Figure 6:**
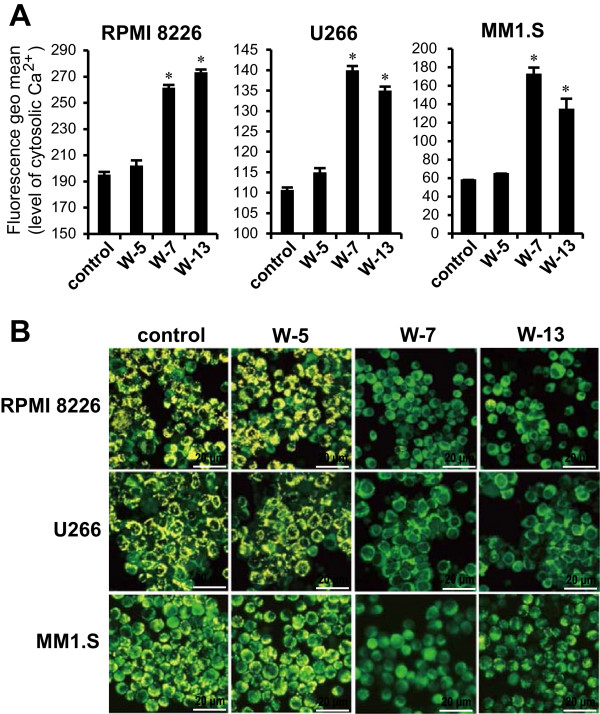
**Effects of CaM antagonists on intracellular Ca** ^**2+**^ **levels and mitochondrial membrane potential. (A)** Cells were loaded with 4 μM Fluo-4/AM dye for 1 h, after which the dyed cells were incubated with CaM antagonists (60 μM) for 30 min. The intracellular Ca^2+^ levels were analyzed by flow cytometry. Data are expressed as means ± standard deviations. *P <0.01 for the comparison with control cells. **(B)** Cells were pretreated with JC-10 dye for 30 min, and subsequently incubated with CaM antagonists (60 μM) for 1 h. The cells were then visualized under a fluorescence microscope to visualize the yellow fluorescent aggregation that indicates high mitochondrial membrane potential which is distinct from the green fluorescent monomer observed at a low mitochondrial membrane potential.

**Figure 7:**
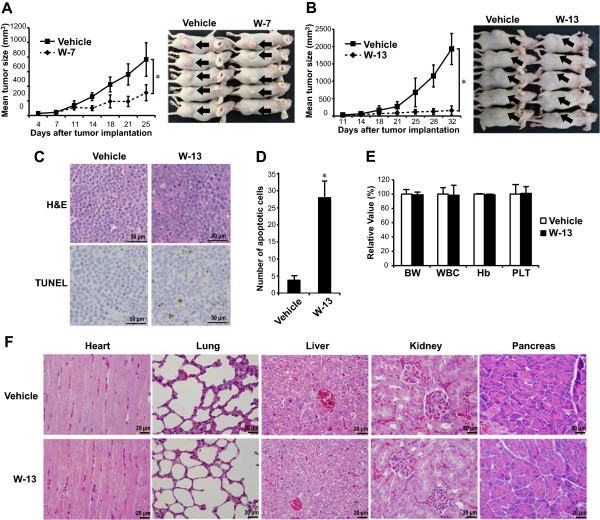
**In vivo antitumor effects of CaM antagonists in a murine multiple myeloma xenograft model.** RPMI 8226 cells were implanted subcutaneously into the flanks of nude mice. Seven days later, 3 mg/kg of CaM antagonists were injected intraperitoneally on 5 days in per week. The tumor sizes were measured twice weekly. The comparison groups were the vehicle (H_2_O) vs. W-7 group **(A)** and the vehicle (PBS) vs. W-13 group **(B)**. The photographs show representative mice, and arrows indicate the tumors. Tumor tissue sections from the mice treated with vehicle or W-13 were stained with hematoxylin/eosin (H&E) or terminal transferase dUTP nick-end labeling (TUNEL) staining **(C)**. TUNEL-positive apoptotic cells were counted in 10 random high power fields **(D)**. Complete blood count and body weight (BW) were also examined in the mice exposed to vehicle or W-13 **(E)**. Data are from five independent animals and are expressed as the mean ± standard deviation. *P <0.01 compared with vehicle. Histology sections of selected organs from the mice treated with vehicle or W-13 were stained with H&E, and representative tissue section of each organ was shown **(F)**.

To explore whether CaM antagonists might act as potential therapeutic agents against MM, we first confirmed protein expression of CaM in the MM cell lines RPMI 8226, U266, MM1.S, MM1.R, KMS-5, KMS-12BM, and NCI-H929 by western blot analysis (Figure 1A), and then determined the effects of the naphthalenesulphonamide derivatives W-7, W-13, and W-5 (a weaker antagonist for CaM used as a negative control for W-7) on the growth of these cell lines. The cells were cultured for 24 h in the presence or absence of CaM antagonists and assessed, using the WST-8 assay. As shown in Figure 1B, W-7 and W-13 inhibited the proliferation of all MM cell lines in a dose-dependent manner. The 50% growth inhibition (IC_50_) values of W-7 and W-13 ranged from approximately 45–60 μM and 30–45 μM, respectively, and W-13 more efficiently inhibited cell proliferation than an identical concentration of W-7. W-5 had little effect on MM cell proliferation.

### Calmodulin antagonists induce G1 phase cell cycle arrest

To determine the mechanisms by which CaM antagonists inhibited MM cell proliferation, we first investigated the effects of CaM antagonists on cell cycle progression. RPMI 8226, U266, and MM1.S cells were treated with CaM antagonists (40 μM). After 24 h, the cells were analyzed by flow cytometry. Treatment with W-7 and W-13 increased the percentage of cells in the G0/G1 phase of the cell cycle and reduced the percentage of cells in the S phase (Figure 2). Treatment with W-5 had no significant effect on the cell cycle compared with the control.

### Effects of CaM antagonists on the expression of cell cycle regulatory proteins in MM cells

To study the molecular mechanism of G0/G1 phase cell cycle arrest induced by CaM antagonists, the expression of various cell cycle-related proteins in MM cells was examined by western blotting after the cells had been treated with CaM antagonists (40 μM) for 24 h. As shown in Figure 3, cyclin D1 expression was reduced following treatment with W-7 and W-13 in U266 cells; RPMI 8226 and MM1.S cells lack cyclin D1 [16]. cyclin D2 and cyclin E1 protein expression was decreased in all cell lines. In RPMI 8226 cells, CDK2, CDK4 and CDK6 expression was decreased in response to W-7 and W-13. In U266 cells, CDK2 and CDK4 expression but not CDK6 expression was decreased. In MM1.S cells, CDK2 and CDK6 expression but not CDK4 expression was decreased. p21^cip1^ protein expression was decreased in all cell lines. However, the expression levels of p27^kip1^ and p53 were unaffected. The levels of phosphorylated Rb were reduced in RPMI 8226 and MM1.S cells; as some U266 clones, including ours, exhibit loss of Rb expression [17]. The same pattern was seen after an incubation period of 12 h (data not shown).

### Calmodulin antagonists induce caspase activation and apoptosis in MM cells

We next studied whether CaM antagonists might also induce apoptosis in MM cells. RPMI 8226, U266 and MM1.S cells were treated with CaM antagonists (60 μM) for 24 h, double-stained with annexin-V and propidium iodide, and analyzed by flow cytometry. The results demonstrated that W-7 and W-13 induced early and late apoptosis in all MM cell lines (Figure 4).

To explore whether CaM antagonists could induce apoptosis through caspase-dependent mechanisms, we performed western blot analysis to examine caspase activation and poly (ADP-ribose) polymerase (PARP) cleavage. RPMI 8226, U266 and MM1.S cells were treated with CaM antagonists (60 μM) for 24 h. The western blot results indicated caspase-3 and caspase-9 activation and PARP cleavage in W-7 and W-13-treated RPMI 8226 cells. However, these treatments had no significant effects on caspase-8 and caspase-7 activation. In U266 and MM1.S cells, caspase-8 and caspase-7 activation were also observed in addition to caspase-3 and caspase-9 activation and PARP cleavage (Figure 5A). The same trend, albeit less dramatic, was seen after an incubation period of 12 h (data not shown).

### CaM antagonists promote ERK1/2 phosphorylation in all MM cells and inhibit STAT3 phosphorylation in U266 and MM1.S cells

Next, we used western blotting to explore intracellular signaling proteins associated with cell survival in MM cells. The cells were treated with CaM antagonists (60 μM) for 24 h. The western blot analysis revealed that treatment with W-7 and W-13 promoted ERK1/2 phosphorylation in all cell lines and inhibited STAT3 phosphorylation in U266 and MM1.S cells but not in RPMI 8226 cells. We also examined the levels of Akt and phospho-Akt in MM cells treated with CaM antagonists but found no significant differences in their levels (data not shown).

### Calmodulin antagonists elevate intracellular Ca^+2^ levels and induce mitochondrial membrane potential depolarization

To further evaluate the molecular mechanism that induced caspase-dependent apoptosis, we examined intracellular Ca^2+^ levels and mitochondrial membrane potential depolarization in response to treatment with CaM antagonists. MM cells that had been pretreated with Fluo-4/AM were incubated with or without CaM antagonists (60 μM) for 30 min and analyzed by flow cytometry. Treatment with W-7 and W-13 increased the intracellular Ca^2+^ levels in all cell lines (Figure 6A). The mitochondrial membrane potential was also assessed using the JC-10 dye. Both W7 and W-13 induced mitochondrial membrane potential depolarization in all evaluated cell lines (Figure 6B).

### Calmodulin antagonists reduce tumor growth rates in murine xenograft models

We next investigated the in vivo efficacy of CaM antagonists in a MM xenograft mouse model. RPMI 8226 cells were inoculated subcutaneously into the flank of each BALB/c *nu* mouse. After the appearance of measurable tumors, the mice were divided into two comparison groups: the vehicle (H_2_O) vs. W-7 (3 mg/kg in H_2_O) group and the vehicle (PBS) vs. W-13 (3 mg/kg in PBS) group. In the vehicle vs. W-7 group, we ended the experiments on day 25 because of tumor ulceration in the vehicle-treated mice, and in the vehicle vs. W-13 group, we ended the experiments on day 32 because the tumor diameters reached 2 cm. Both W-7 and W-13 inhibited tumor growth relative to their respective vehicles (Figure 7A and B).

To examine the in vivo cytotoxic effects of CaM antagonists, we performed H&E staining and a TUNEL apoptosis assay in tumor tissues excised from the mice treated with vehicle (PBS) or W-13. Although H&E staining showed that residual MM cells remained in the W-13-treated tumors, TUNEL-positive apoptotic cells were significantly increased relative to the vehicle-treated tumors (Figure 7C and D). We also examined the adverse effects of W-13. No significant changes in complete blood count, body weight, or other appearances of toxicity were observed in the animals (Figure 7E). In addition, pathological screening of the H&E sections of heart, lung, liver, kidney, and pancreas showed no apparent changes in the W-13-treated animals, except for in one mouse who had slight inflammation in the pancreas (Figure 7F).

## Discussion

In the present study, we have shown that CaM antagonists inhibited MM cell proliferation in vitro and in vivo, and to elucidate the mechanisms of action of CaM antagonists, we have revealed two cellular and molecular mechanisms: induction of cell cycle arrest and induction of apoptosis.

Our cell cycle analysis revealed that CaM antagonists could markedly induce G1 phase arrest in MM cells. Cell cycle progression is driven by the activation of specific cyclin– CDK complexes at different intervals. The formation of complexes between D-type cyclins and CDK4 and CDK6 is required for G1 phase progression [18]. CaM has been shown to be essential for CDK4 activation and nuclear cyclin D1–CDK4 complex accumulation during the G1 phase [19], and recent research has shown that CaM competes with F-box protein FBXL-2, which promotes ubiquitination and degradation of cyclin D2 [20]. It therefore seems reasonable to suppose that CaM antagonists would inhibit the activities of cyclin D–CDK4/CDK6 complexes in MM cell lines.

We also revealed that cyclin E–CDK2 complex formation was downregulated in response to treatment with CaM antagonists. Complex formation between cyclin E and CDK2 is rate limiting and essential for S phase entry [21]. Human cyclin E genes have been reported to contain a CaM-binding motif, and CaM has a direct stimulatory effect on cyclin E–CDK2 [22]. Furthermore, the downregulated CDK2 expression was previously observed in response to treatment with the CaM antagonist W-13 in a T lymphocyte-based experiment [23]. Together, these reports suggest that CaM is essential for the activation of cyclin E–CDK2 complexes.

p21^cip1^ is a potent cyclin-dependent kinase inhibitor that binds to and inhibits the activities of CDKs and thus functions as a regulator of cell cycle progression through the G1 and S phases [24]. CaM antagonists have been shown to induce sustained ERK1/2 activation and p21^cip1^ overexpression [25]. Sustained ERK pathway activation induces cell cycle arrest, whereas transient ERK activation is a common feature of cell proliferation in many systems [26]. This dual effect of ERK1/2 on cell proliferation has been shown to depend on p21^cip1^ expression [27, 28]. The ERK pathway also induces the expression of the positive cell cycle regulator cyclin D1. The inability to induce cell proliferation following a strong ERK activation is associated with a lack of cyclin D1 induction [29]. In agreement with that earlier finding, our results showed that CaM antagonist-induced ERK1/2 activation correlated with p21^cip1^ overexpression in the absence of cyclin D1 upregulation.

Another mechanism by which CaM antagonists inhibit MM cell proliferation is caspase-dependent apoptosis. Apoptotic pathways can be divided into those that involve extrinsic death receptor signaling with the activation of the initiator caspase-8 and those that involve intrinsic mitochondrial damage with the activation of the initiator caspase-9 [30]. Our data revealed that CaM antagonists induced the activation of caspase-8 and caspase-7 in U266 and MM1.S cells but not in RPMI 8226 cells and the activation of caspase-9 and caspase-3 in all MM cell lines, suggesting that CaM antagonists induced apoptosis via the extrinsic pathway in U266 and MM.S cells and via the intrinsic pathway in all cell lines.

Recent studies have suggested that frequent activation of STAT3 signaling provides a survival advantage to MM cells [31–33] and that the STAT3 pathway mediates the induction of antiapoptotic proteins such as Mcl-1, Bcl-2, and Bcl-xL. Of these antiapoptotic proteins, Mcl-1 is an essential survival factor [34]. Our data revealed that STAT3 phosphorylation was inhibited in response to treatment with CaM antagonists, thus leading to reduced Mcl-1 protein expression in U266 and MM1.S cells but not in RPMI 8226 cells. We therefore assumed that the differences between the MM cell lines in terms of the extrinsic apoptotic pathway resulted from the inhibition of STAT3 phosphorylation. The mechanism associated with the differences in STAT3 inactivation in the different cell lines is unclear and will require further investigation.

The elevated intracellular Ca^2+^ level and mitochondrial membrane potential depolarization observed in our research comprise still another identified molecular event identified associated with CaM antagonist-mediated apoptosis. Naphthalenesulphonamide derivative CaM antagonists have been shown to induce increases in the intracellular Ca^2+^ levels in several cell types [35–37]. A sustained increases in the cytosolic Ca^2+^ levels leads to the collapse of the mitochondrial membrane potential via Ca^2+^ overloading of the mitochondria [38]. After mitochondrial depolarization, cytochrome *c* is released to accumulate in the cytoplasm, followed by caspase-9 and caspase-3 activation, which cleaves PARP and ultimately leads to apoptosis [28].

Finally, CaM antagonists were well tolerated and very effective in an in vivo murine MM model, as evidenced by the inhibition of MM tumor growth in mice injected intraperitoneally with CaM antagonists. In particular, W-13 inhibited tumor growth more effectively than W-7 at the same dosage. Surprisingly, relatively low dose of W-13 was very effective compared with the high IC_50_ value in vitro. We assume that the efficacy in vivo is partly due to the fact that W-7 and W-13 possess highly water-soluble and cell-permeable properties and seem to have high bioavailability. W-7 and W-13 have been reported to inhibit the formation of bovine erythroid colonies [39]. However, no significant change in complete blood count was observed in the W-13-treated animals. Pathological screening of the H&E sections of heart, lung, liver, kidney, and pancreas showed no apparent changes in the W-13-treated animals, except for in one mouse who had slight inflammation in the pancreas. This inflammation may be associated with the intraperitoneal injection of the drugs.

It is important to mention that there are potential off-target effects of CaM antagonists, and some pharmacological effects of CaM antagonists might not be mediated solely via the direct inhibition of CaM. In addition, our animal model reflects a plasmacytoma model and represents a minority of clinical myeloma cases. Therefore, further experimental studies are needed to address these limitations.

## Conclusions

We have shown that CaM antagonists induce cell cycle arrest, induce apoptosis via caspase activation, and inhibit tumor growth in a murine MM model. These results raise the possibility that inhibition of CaM might be a useful therapeutic strategy for the treatment of MM.
